# Exploring the link between a novel approach for computer aided lung sound analysis and imaging biomarkers: a cross-sectional study

**DOI:** 10.1186/s12931-024-02810-5

**Published:** 2024-04-24

**Authors:** Eline Lauwers, Toon Stas, Ian McLane, Annemiek Snoeckx, Kim Van Hoorenbeeck, Wilfried De Backer, Kris Ides, Jan Steckel, Stijn Verhulst

**Affiliations:** 1 Laboratory of Experimental Medicine and Pediatrics and member of Infla-Med Research Consortium of Excellence, University of Antwerp, Wilrijk, Belgium; 2 CoSys-Lab Research Group, University of Antwerp and Flanders Make Strategic Research Center, Wilrijk, Lommel, Belgium; 3 Sonavi Labs, Baltimore, MD USA; 4 Department of Radiology, Antwerp University Hospital, Edegem, Belgium; 5 Department of Pediatrics, Antwerp University Hospital, Edegem, Belgium; 6 Faculty of Medicine and Health Sciences, University of Antwerp, Wilrijk, Belgium; 7 Fluidda NV, Kontich, Belgium; 8 MedImprove BV, Kontich, Belgium; 9 Department of Electrical and Computer Engineering, Johns Hopkins University, Baltimore, MD USA

**Keywords:** Cystic fibrosis, Digital lung auscultation, Computer aided lung sound analysis, Chest computed tomography, Functional respiratory imaging

## Abstract

**Background:**

Computer Aided Lung Sound Analysis (CALSA) aims to overcome limitations associated with standard lung auscultation by removing the subjective component and allowing quantification of sound characteristics. In this proof-of-concept study, a novel automated approach was evaluated in real patient data by comparing lung sound characteristics to structural and functional imaging biomarkers.

**Methods:**

Patients with cystic fibrosis (CF) aged > 5y were recruited in a prospective cross-sectional study. CT scans were analyzed by the CF-CT scoring method and Functional Respiratory Imaging (FRI). A digital stethoscope was used to record lung sounds at six chest locations. Following sound characteristics were determined: expiration-to-inspiration (E/I) signal power ratios within different frequency ranges, number of crackles per respiratory phase and wheeze parameters. Linear mixed-effects models were computed to relate CALSA parameters to imaging biomarkers on a lobar level.

**Results:**

222 recordings from 25 CF patients were included. Significant associations were found between E/I ratios and structural abnormalities, of which the ratio between 200 and 400 Hz appeared to be most clinically relevant due to its relation with bronchiectasis, mucus plugging, bronchial wall thickening and air trapping on CT. The number of crackles was also associated with multiple structural abnormalities as well as regional airway resistance determined by FRI. Wheeze parameters were not considered in the statistical analysis, since wheezing was detected in only one recording.

**Conclusions:**

The present study is the first to investigate associations between auscultatory findings and imaging biomarkers, which are considered the gold standard to evaluate the respiratory system. Despite the exploratory nature of this study, the results showed various meaningful associations that highlight the potential value of automated CALSA as a novel non-invasive outcome measure in future research and clinical practice.

**Supplementary Information:**

The online version contains supplementary material available at 10.1186/s12931-024-02810-5.

## Background

Respiratory sounds contain valuable information for the diagnosis and progression of pulmonary diseases. Standard auscultation, however, is a subjective process depending on the examiner’s hearing and experience. In addition, no permanent records can be made and breathing patterns or changes over time cannot be quantified. Over the last decades electronic stethoscopes and digital signal processing techniques have been developed to overcome these limitations. Computer Aided Lung Sound Analysis (CALSA) provides objective information and can be used as a noninvasive, low-cost, bedside measure. Various methods for computational analysis have been proposed in previous research, ranging from classical signal-processing methods to data-science methods that build upon features to create classifiers using deep learning strategies [1, 2]. To date, most automated algorithms have been directed at the presence of one or more adventitious respiratory sounds in the patient’s recording to support physicians in making an accurate diagnosis. By contrast, continuous parameters to indicate the severity level of a patient’s disease are lacking in current literature. These continuous output values could facilitate the comparison of recordings and monitor disease progression over time. In this paper, a novel automated analysis is performed to characterize respiratory sounds in subjects with cystic fibrosis (CF), focusing on automated respiratory cycle detection, inspiratory and expiratory sound power, crackle and wheeze analysis. As this approach is novel to the field, a better understanding of the relation between the obtained respiratory sound characteristics and existing outcome measures to evaluate respiratory health in real patient data is needed. At present, Computed Tomography (CT) is the gold standard to evaluate structural airway and lung parenchymal abnormalities in CF, such as mucus plugging, air trapping, bronchiectasis and bronchial wall thickening [3]. In addition, CT datasets offer the potential of data analysis with different algorithms. Functional Respiratory Imaging (FRI) is a 3D-imaging technique combined with Computational Fluid Dynamics (CFD), providing patient-specific information on a lobar level related to airflow and airway resistance [4]. The generation and transmission of lung sounds can be linked to airflow, geometry of the bronchial tree, narrowing of the airways, airway wall stability, density of the lung parenchyma, etc [5]. Therefore, we would expect from a physiological point of view that results obtained by automated CALSA will be associated with certain regional imaging parameters. A search of the literature revealed no previous studies that explored the association between auscultatory findings and CT imaging. Therefore, this proof-of-concept aimed to assess to what extent digital lung sounds using an automated approach are related to structural and functional imaging biomarkers in subjects with CF lung disease. Additionally, associations between lung sound characteristics and spirometry as a classical pulmonary function test were explored as well.

## Methods

### Study population

In this prospective cross-sectional study, patients were recruited at the Antwerp University Hospital from May 2017 to January 2020. All patients that were scheduled for a chest CT during this enrollment period were considered for participation to limit additional radiation exposure. Indications to perform a chest CT included a control CT to follow up on disease progression, or participation in another clinical study requiring a chest CT. When subjects were recruited participating in another study, they needed to be on a stable treatment regimen for the past four weeks. It is important to note that all study assessments were performed according to the same protocols as outlined below and they were all organized on the same day. Subjects were eligible for inclusion if they met the following criteria: CF diagnosis, age > 5 years, and clinically stable at inclusion. Patients were considered clinically stable if they were on a stable regimen for CF medication for four weeks prior to the study assessments. Patients with cognitive impairment were excluded. Prior to participation, signed informed consent was obtained from all subjects and their parents/guardians in case the subject was a minor. The study was approved by the Ethics Committee of the Antwerp University Hospital (BE300201630558).

### Study assessments

#### Lung function, demographic and anthropometric data

Demographic data, including date of birth, sex and ethnicity were collected first. Body length and weight were measured to calculate Body Mass Index (BMI). Spirometry was performed using the Jaeger Masterscreen PFT (CareFusion, San Diego, USA) or the Spirostik (Geratherm Respiratory, Germany) according to ERS standards [6].

#### CT imaging

Unenhanced chest CT scans were acquired with a GE VCT LightSpeed 64-slice scanner at two breathing levels, Total Lung Capacity (TLC) and Functional Residual Capacity (FRC), monitored by a pneumotachograph. Specific settings of the scanning protocol can be found in the supplementary material. Structural abnormalities were quantified using the CF-CT score by two independent observers blinded to subject ID, of whom one experienced chest radiologist and one certified researcher [7, 8]. Following components were scored on a lobar level: bronchiectasis (0–12), bronchial wall thickening (0–9), mucus plugging (0–6), parenchymal abnormalities (0–9), and air trapping (0-4.5), which resulted in an overall severity score (0-40.5).

#### FRI

A 3D reconstruction of the airways and lung lobes is performed via segmentation of the CT scans. Images at two lung levels, TLC and FRC, allow the assessment of geometric changes over the breathing cycle. After segmentation and postprocessing, the models were used for CFD to simulate airflow by solving Reynolds-averaged Navier-Stokes equations to add a functional element to the static images. The following parameters were extracted from the analysis: airway volume, air trapping and airway resistance. Airway resistance within the patient-specific airway model was calculated by CFD taking into account the relative internal airflow distribution to the respective lung lobes. A more detailed description of the FRI technology can be found in the supplementary material and in previously published work [4, 9].

#### CALSA

Respiratory sounds were recorded using a digital stethoscope (Thinklabs One, Thinklabs Medical LLC) at six chest locations: two posterior basal, two anterior (2nd intercostal space, mid-clavicular line) and two lateral (4th-5th intercostal space, mid-axillary line). Each recording was made for 25–30 s with the patient in a sitting position, while breathing through the mouth. The stethoscope was connected to the sound card of a laptop with commercial software (Audacity, version 2.1.2.). Respiratory sounds were acquired in accordance with the CORSA guidelines for short-term acquisition [10].

All CALSA analyses were performed in MATLAB (The MathWorks, Inc.). In general, automated CALSA consisted of four main components: pre-processing and denoising steps to enhance the quality of the recordings, wavelet packet decomposition to separate discontinuous adventitious sounds from the signal, respiratory cycle extraction, and crackle peak detection. A detailed outline of the analysis was published by McLane et al. [11], and a schematic overview of the processing steps can be found in the supplementary material (Figure E1). A semi-automated approach was applied for the respiratory cycle detection to allow visual corrections if needed. The signal power during in- and expiration was used to calculate the expiration-to-inspiration (E/I) ratio for different frequency bands, i.e. 100–200 Hz, 200–400 Hz, 400–800 Hz, and 800–1600 Hz. The average crackle count was determined per respiratory phase. Apart from the approach proposed by McLane et al., wheeze analysis was performed to provide a more comprehensive overview of the most prevalent lung sound characteristics. A Fast Fourier Transform approach was used to analyze wheeze segments as described by Nabi et al. [12]. Parameters of interest were: mean frequency, frequency quartiles in a normalized power spectrum, and wheeze occupation rate.

### Statistics

Statistical analyses were performed in R for statistical computing (version 4.1.0, R Core Team 2021, Austria), using the following R packages: ‘stats’, ‘irr’, ‘blandr’, ‘nlme’, and ‘ggplot2’. Histograms and QQ plots were computed to evaluate the distribution of the data. Mathematical transformations to the variables were performed to match normal distribution, if applicable. Interobserver reliability of the CF-CT scoring was examined by Bland-Altman plots and intraclass correlation coefficients (ICCs) using a two-way mixed-effects model for average measures [13]. To investigate the association between lung sound characteristics and imaging parameters per zone, linear mixed-effects models were computed with subject and zone as random effects. The left and right anterior recordings were compared to the respective upper lobes, posterior recordings to the respective lower lobes, and the right lateral recording was compared to the right middle lobe. Similar mixed-effects models with only subject as a random effect were computed for the comparison between average values of the six recordings and spirometry. No power calculation was performed, since no prior information was available on expected sample variance. Statistical significance was accepted when *P* < 0.05.

## Results

### Study population

Since all eligible CF patients scheduled for a chest CT during the enrollment period were considered for participation, some patients entered the study more than once. None of the patients underwent the study assessments more than twice, and the period between repeated measurements ranged from 3 months to 2 years. As explained in the statistics, dependency of the data was accounted for using mixed effects models with subject ID as a random effect.

In total, 41 study assessments were performed in 26 participants. Prior to further analyses, four study assessments were excluded due to bad quality of the recordings. These recordings were not considered clinically relevant as electronical interference, friction and/or ambient noise hampered the recognition of any breath sounds. As a result, 37 study assessments were included from 25 (16 M/9F) participants with a median age of 22 [6;46] years, a median BMI of 20.9 [12.2;27.0] kg/m^2^ and a median forced expiratory volume in one second (FEV_1_) of 68 [26;123] % predicted. Twelve patients received CFTR modulator therapy (Orkambi^®^) at the time of the study assessments.

The interobserver variability analysis of the CF-CT score showed moderate to excellent absolute agreement between observers with ICCs between 0.41 (bronchial wall thickening) and 0.91 (bronchiectasis). These results are consistent with previous research concerning visual scoring methods [14]. A comprehensive overview of all ICCs and Bland-Altman plots can be found in the supplementary material (Table E1, Figures E2-7).

### Automated CALSA

Six recordings per study assessment were acquired, resulting in a total of 222 recordings to analyze. In 68/222 (31% of total) recordings adjustments were made to the respiratory cycle annotation, and 18/222 (8% of total) required substantial changes of the cycle times. Median values of the frequency band analysis and crackle detection are shown in Table 1. Although it is widely recognized that age and body height influence the intensity and frequency of normal breath sounds [15, 16], neither age nor body height were significantly associated with any of the E/I ratios. In only one recording wheezing was present. Two expiratory wheezes were detected with a wheeze occupation rate of 22.8 and 3.4% and a mean frequency of 206.2 and 181.1 Hz, respectively. Wheeze characteristics were not considered for additional statistical analyses, since no cross-sectional comparison could be made.

**Table 1:** CALSA results (*n* = 222)

E/I ratio	100–200 Hz	0.25 [0.02; 1.17]
	200–400 Hz	0.17 [< 0.01; 2.03]
	400–800 Hz	0.24 [< 0.01; 5.37]
	800–1600 Hz	0.36 [0.01; 6.71]
Crackle count	Full cycle	6.00 [0.00; 24.00]
	Inspiration	2.27 [0.00; 13.50]
	Expiration	3.43 [0.00; 13.00]

Data are presented as median [range]. E/I ratio, expiration-to-inspiration signal power ratio.

### Automated CALSA versus CF-CT

**Fig. 1:**
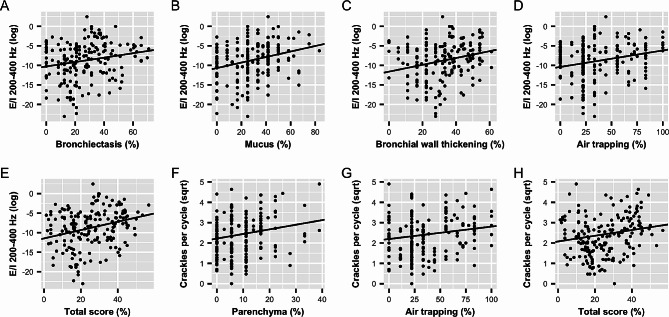
**A-H.** Lung sound characteristics vs. CF-CT scores with the regression lines of the mixed-effects models. E/I, expiration-to-inspiration signal power ratio

The linear mixed-effects models showed multiple significant associations between lung sound characteristics and CF-CT scores on a lobar level. Of the E/I ratios, the power ratio between 200 and 400 Hz showed a significant positive association with the total CF-CT score (*p* < 0.001) and bronchiectasis (*p* = 0.013), mucus plugging (*p* = 0.003), bronchial wall thickening (*p* = 0.002), and air trapping (*p* = 0.003). Significant associations were also found for other frequency ranges, showing that increased E/I ratios were related to the extent of air trapping, bronchial wall thickening and the CF-CT total score. Considering the crackle analysis, the average number of crackles was related to the total score (*p* = 0.025), the extent of parenchymal abnormalities (*p* = 0.006) and air trapping (*p* = 0.030). Inspiratory crackles showed more significant associations, as this feature was also related bronchiectasis (*p* = 0.038) and mucus plugging (*p* = 0.036). By contrast, the number of expiratory crackles could only be associated with the extent of parenchymal abnormalities (*p* = 0.003). An overview of all pairwise comparisons can be found in Table 2 and significant associations are presented as scatterplots in Fig. 1A-H.

**Table 2:** Automated CALSA vs. CF-CT

	CF-CT score (%)					
	BE	Mucus	BWT	Parenchyma	Air trapping	Total
**E/I ratio (log)**						
100–200 Hz	*P* = 0.146	*P* = 0.076	*P* = 0.052	*P* = 0.328	↗, *P* = 0.041*	*P* = 0.073
200–400 Hz	↗,*P* = 0.013*	↗, *P* = 0.003*	↗,*P* = 0.002*	*P* = 0.329	↗,*P* = 0.003*	↗,*P* < 0.001*
400–800 Hz	*P* = 0.078	*P* = 0.056	↗,*P* = 0.008*	*P* = 0.584	↗,*P* = 0.038*	↗,*P* = 0.015*
800–1600 Hz	*P* = 0.053	*P* = 0.126	↗,*P* = 0.031*	*P* = 0.982	↗,*P* = 0.022*	↗,*P* = 0.027*
**Crackle count (sqrt)**						
Full cycle	*P* = 0.090	*P* = 0.242	*P* = 0.209	↗,*P* = 0.006*	↗,*P* = 0.030*	↗,*P* = 0.025*
Inspiration	↗,*P* = 0.038*	↗,*P* = 0.036*	*P* = 0.082	↗,*P* = 0.035*	↗,*P* = 0.021*	↗,*P* = 0.008*
Expiration	*P* = 0.173	*P* = 0.900	*P* = 0.358	↗,*P* = 0.003*	*P* = 0.070	*P* = 0.078

*P*-values of the mixed-effects models are presented. Abbreviations: ↗, positive association; BE, bronchiectasis; BWT, bronchial wall thickening; E/I ratio, expiration-to-inspiration signal power ratio; sqrt, square root; *, *P* < 0.05.

### Automated CALSA versus FRI

**Fig. 2:**
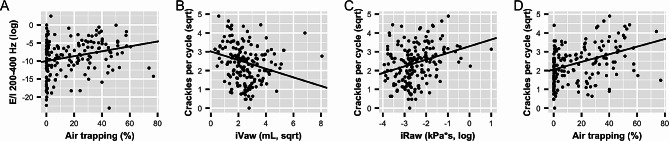
**A-D.** Lung sound characteristics vs. FRI parameters with the regression lines of the mixed-effects models. E/I, expiration-to-inspiration signal power ratio; iVaw, airway volume; iRaw, airway resistance

When comparing FRI parameters (i.e. airway volume, airway resistance and air trapping) to E/I ratios, only a significant positive association was found between E/I 200–400 Hz and air trapping (*p* = 0.007), and between E/I 800–1600 and airway volume (*p* = 0.017). On the other hand, the average number of crackles was negatively associated with the total airway volume per lung lobe (*p* = 0.002) and positively associated with airway resistance and air trapping (both *p* < 0.001). When considering the number crackles during inspiration and expiration separately, similar associations could be found. Results of the mixed-effects models comparing automated CALSA to FRI are shown in Table 3; Fig. 2A-D.

**Table 3:** Automated CALSA vs. FRI

	FRI		
	iVaw (mL)	iRaw (kPa*s, log)	Air trapping (%)
**E/I ratio (log)**			
100–200 Hz	*P* = 0.318	*P* = 0.337	*P* = 0.061
200–400 Hz	*P* = 0.067	*P* = 0.897	↗,*P* = 0.007*
400–800 Hz	*P* = 0.447	*P* = 0.912	*P* = 0.075
800–1600 Hz	↗,*P* = 0.017*	*P* = 0.332	*P* = 0.082
**Crackle count (sqrt)**			
Full cycle	↘,*P* = 0.002*	↗,*P* < 0.001*	↗,*P* < 0.001*
Inspiration	↘,*P* = 0.001*	↗,*P* = 0.003*	↗,*P* < 0.001*
Expiration	↘,*P* = 0.010*	↗,*P* = 0.002*	↗,*P* = 0.001*

*P*-values of the mixed-effects models are presented. Abbreviations: ↗, positive association; ↘, negative association; E/I ratio, expiration-to-inspiration sound power ratio; iVaw, airway volume; iRaw, airway resistance; sqrt, square root; *, *P* < 0.05.

### Automated CALSA versus spirometry

To compare lung sounds to spirometry, the average of each parameter was calculated across all six chest locations. No significant correlations were found between E/I ratios and spirometry. The average number of inspiratory crackles showed a significant negative association with FEV_1_%pred (*p* = 0.028) and MEF_25 − 75_%pred (*p* = 0.011). Results can be found in Table 4.

**Table 4:** Automated CALSA vs. Spirometry

	Spirometry (% predicted)				
	FEV_1_	FVC	PEF	MEF_25 − 75_ (log)	MEF_25_ (log)
**E/I ratio (log)**					
100–200 Hz	*P* = 0.160	*P* = 0.475	*P* = 0.816	*P* = 0.163	*P* = 0.203
200–400 Hz	*P* = 0.302	*P* = 0.705	*P* = 0.808	*P* = 0.232	*P* = 0.242
400–800 Hz	*P* = 0.583	*P* = 0.339	*P* = 0.116	*P* = 0.780	*P* = 0.876
800–1600 Hz	*P* = 0.329	*P* = 0.877	*P* = 0.769	*P* = 0.242	*P* = 0.274
**Crackle count (sqrt)**					
Full cycle	*P* = 0.282	*P* = 0.756	*P* = 0.621	*P* = 0.109	*P* = 0.331
Inspiration	↘, *P* = 0.047*	*P* = 0.214	*P* = 0.191	↘, *P* = 0.013*	*P* = 0.077
Expiration	*P* = 0.808	*P* = 0.632	*P* = 0.825	*P* = 0.445	*P* = 0.793

*P*-values of the mixed-effects models are presented. Abbreviations: ↗, positive association; ↘, negative association; E/I ratio, expiratory/inspiratory ratio; FEV_1_, forced expiratory volume in one second; FVC, forced vital capacity; MEF_25−75_, mean expiratory flow between 25 and 75% of FVC; MEF_25_, maximal expiratory flow when 25% of FVC remains to be exhaled; sqrt, square root; *, *P* < 0.05.

## Discussion

In this proof-of-concept, a novel approach for automated CALSA was evaluated in real patient data. To obtain more insight into the clinical value of respiratory sound analysis, the relationship between multiple CALSA parameters and imaging biomarkers was investigated in a heterogeneous group of CF patients. Results of the statistical analysis showed significant associations between E/I power ratios within several frequency ranges and structural abnormalities shown on CT. The average number of crackles was also associated with multiple structural abnormalities on CT and airway resistance determined by FRI.

The intensity of normal breath sounds peaks between 100 and 200 Hz with an energy drop above 300 Hz. Generally, breath sounds are audible at the chest during inspiration, while the expiratory sound has a much lower intensity [15, 17]. In our study, in- and expiratory sound power could not be considered individually, as the digital stethoscope applies a build-in algorithm to amplify the sound of the recording depending on the overall intensity of the signal. Therefore, only the power ratios were retained to allow comparison between recordings. An increased E/I ratio can either be related to a decreased inspiratory sound or an increased expiratory sound. As sound intensity is directly related to respiratory flow, diminished inspiratory breath sounds could indicate poor ventilation of the respective lung region [16, 18]. On the other hand, a relative increase in expiratory sound, often named ‘bronchial breathing’, has been reported previously to be related to morphological changes of the airways and lung parenchyma resulting from bronchoconstriction, airway inflammation and/or consolidation [17, 19, 20]. Therefore, it was expected that increased E/I ratios within the frequency range of normal breath sounds could be related to pulmonary disease. Although the intensity of normal breath sounds is the highest between 100 and 200 Hz, these sounds are mixed with cardiovascular and muscle sounds [16], which makes it more difficult to distinguish between sounds. This could explain why the E/I ratios in higher frequency ranges showed more associations with imaging biomarkers compared to E/I 100–200 Hz. Our results suggest that E/I 200–400 Hz is the most promising parameter of the frequency band analysis to indicate the severity level of CF lung disease, as most associations were found within this range. This ratio has already been pointed out in previous research to be an indicator of airway narrowing and inflammation in asthma [21, 22]. As the majority of the CF-CT subscores were associated with E/I 200–400 Hz, the power ratio cannot be used to discriminate between different pulmonary manifestations. However, this could be due to the interdependency of abnormalities, e.g. bronchiectasis will most often be accompanied by increased mucus plugging. Only the extent of parenchymal abnormalities was not associated with E/I 200–400 Hz, although a significant result would have been expected due to changes in sound transmission following consolidation or atelectasis. The interdependency of the CF-CT subscores was verified by computing Spearman correlations between these scores, considering only the first scan of the subjects. As such, the highest correlations were found between bronchiectasis and mucus (*R* = 0.74, *p* < 0.05), between bronchiectasis and air trapping (*R* = 0.74, *p* < 0.05), and between bronchiectasis and bronchial wall thickening (*R* = 0.73, *p* < 0.05). Overall, lower correlations were found between parenchymal abnormalities and all other subscores with correlation coefficients ranging from 0.46 to 0.65 (all *p* < 0.05). When considering the mixed effects models of CALSA and air trapping, a difference can be seen in associations with air trapping as reported by CF-CT vs. FRI. Although both endpoints aim to reflect the same respiratory condition, both methods differ substantially. On the one hand air trapping according to CF-CT is a subjective score based on the extent and pattern of this abnormality, while air trapping calculated by FRI is a density measure.

In addition to the frequency band analysis, the average number of crackles also showed several significant associations with structural as well as functional abnormalities. CF lung disease is typically associated with coarse crackles that can be heard during early to mid-inspiration and to a lesser extent throughout expiration [22]. The origin of crackles has been attributed to elastic stress in the airway walls related to sudden opening or closing of collapsed airways, movement of thin secretions and rupture of fluid menisci [17, 23]. Keeping these physiological principles in mind, it is not surprising that especially the number of inspiratory crackles could be related to lung structure and function. The average number of crackles during both in- and expiration were significantly associated with all three FRI parameters: airway volume, airway resistance and air trapping. In contrast to all other imaging biomarkers, a negative association was found with total airway volume per lung lobe. In other words, a decrease in airway volume was associated with a higher number of crackles. A decreased airway volume can result from the presence of mucus, inflammation, and/or bronchoconstriction. Since airway volume determined by FRI only considers intraluminal air, these abnormalities reduce the volume even in enlarged airways due to bronchiectasis. This reasoning is strengthened by the positive relation between the number of crackles and regional airway resistance. In particular, the presence of mucus, inflammation, and bronchoconstriction are expected to result in an increase in airway resistance. However, it’s important to note that airway resistance, as calculated by FRI, is based only on the airways visible on CT, which is limited by the resolution of the scan. The number of generations included in the airway model differ for each individual depending on the point where no distinction can be made anymore between intraluminal and alveolar air.

Although these results are promising and can overall be explained from a physiological point of view, they should be interpreted with caution. An important limitation of research in the field of computerized respiratory sound analysis in general is that no gold standard is available to evaluate new automated approaches. Most algorithms at present are validated against manual annotations, but this subjective method is inevitably associated with considerable inter- and intra-observer variability [24]. In addition, often only a relatively small dataset is feasible to annotate, preventing the algorithms to be generalized to a more heterogeneous group of subjects [25]. For this reason, the approach applied in this study was recently validated by McLane et al. using a large dataset of simulated lung sounds, such that the exact timing of respiratory phases and crackle peaks were known [11]. The performance of the cycle extraction and crackle peak detection was 96% and 95% (expressed as F-scores), respectively. Notwithstanding the advantages of a simulated dataset, real patient data are more complex, which has an impact on the accuracy of the algorithms. Therefore, minor adjustments to respiratory cycle annotations were made as all lung sound parameters depend on the timing of the respiratory phases. Also, a link was found between the quality of the recordings and the number of crackles detected, despite the implementation of a denoising algorithm. Since crackles, friction and motion artifacts are all discontinuous explosive sounds, the latter two features are often incorrectly recognized as crackles. However, it would not have been feasible to manually review the crackle detection due to the resemblance of crackles and friction, the overlap of subsequent crackles and the dominance of louder breath sounds or ambient noise. Fortunately, the number of crackles was related to various pulmonary manifestations, which suggests that the clinical value of the detected crackles was sufficient to overcome the introduced errors. Considering the statistical approach, CALSA endpoints were compared 1-by-1 to imaging endpoints, which resulted in multiple mixed-effects models. The *p*-values were reported for each mixed model individually, but this may have increased the risk for type I errors. An adapted significance level could be applied to compensate for the multiple comparisons by dividing 0.05 by the number of endpoints for CF-CT, FRI and spirometry, respectively. Consequently, mainly the comparisons between E/I 200–400 against the CF-CT scores, and the comparisons of the number of crackles against the FRI endpoints would remain significant.

As this is a proof-of-concept study, various improvements to the methodology are required and a number of questions remain to be answered in future research. First, recordings at six standardized chest locations were performed in accordance with the CORSA guidelines [10]. However, the locations were not ideal for one-on-one comparison with imaging biomarkers on a lobar level. As such, the right lateral recording was matched with the right middle lobe, although the right upper and lower lobe can be auscultated at this point as well. Therefore, it would have been interesting to include additional recordings that better reflect individual lung lobes. Secondly, airflow was not standardized during the recording, while airflow and lung volume have a considerable impact on the generation of normal breath sounds as well as adventitious respiratory sounds [10, 26]. It was decided to perform the recordings during spontaneous tidal breathing, as this approach is the most feasible in clinical practice. Next, we did not include a healthy control group and we were therefore not able to compare our results to a reference population. Nevertheless, a heterogeneous group of patients was included with an FEV_1_ ranging from 26 to 123% predicted, which allowed us to assess lung sound characteristics at different stages of disease. Further work is required to set an upper limit of normal for each parameter, ideally adjusted for different age categories, body height and sex, since these factors are known to influence normal breath sounds [15]. Besides an upper limit of normal, variability over time of the parameters acquired by this novel automated analysis should be verified. This would enable these characteristics to be used as endpoints in research or for clinical follow-up in a later stage. For future research it would be recommended to use an alternative digital stethoscope that does not alter the sound power as mentioned previously. Lastly, only the number of crackles was considered, but it would be interesting to obtain additional information about the characteristics of crackles, such as intensity and coarseness. Characterization of crackles would provide physicians and health providers more insight into the pathophysiological processes that generate these crackles [27].

Overall, this study offers a first step towards clinical validation of the parameters derived from automated CALSA. The continuous output values provide objective and regional information that might contribute to a better understanding of disease severity and progression. As mentioned in the introduction, CT imaging is the gold standard to evaluate the respiratory system, but this measure is only available in a hospital setting and is not suitable for frequent assessment due to the associated radiation dose. Digital auscultation, on the other hand, can be adopted in various settings, since the process is non-invasive, requires only a minimal setup and can be performed without patient cooperation. In this regard, automated CALSA could be a valuable tool for CF, as this population is in need for early and intensive follow-up to minimize the negative consequences related to the vicious cycle of inflammation and infection. Furthermore, automated CALSA could provide additional information to conventional pulmonary function tests (e.g. spirometry) by assessing the lungs on a regional level.

## Conclusions

To conclude, to our knowledge this is the first study that correlated digital lung sound characteristics to imaging biomarkers. Multiple significant associations were found between E/I power ratios, the number of crackles and structural and functional features on a lobar level. Considering the power ratios, an increased E/I ratio at a frequency range of 200–400 Hz appeared to be most clinically relevant due to its association with abnormalities including bronchiectasis, mucus plugging, bronchial wall thickening and air trapping. The number of crackles were, besides structural abnormalities, also related to pulmonary function, such as regional airway resistance, FEV_1_ and MEF_25 − 75_. These results show the potential value of automated CALSA as outcome measure in research and clinical practice. Future research is required to improve the methodology and to determine its role as clinical outcome measure.

### Electronic supplementary material

Below is the link to the electronic supplementary material.

Supplementary Material 1

## Data Availability

All data are summarized within the article and the supplementary material. Individual patient data are not publicly available, but they are available from the corresponding author upon reasonable request.

## Abbreviations

BE: Bronchiectasis
BMI: Body Mass Index
BWT: Bronchial wall thickening
CALSA: Computer Aided Lung Sound Analysis
CF: Cystic fibrosis
CF-CT: Cystic fibrosis – Computed tomography scoring system
CFD: Computational fluid dynamics
CT: Computed tomography
E/I: Expiration-to-inspiration signal power ratio
FEV_1_: Forced expiratory volume in one second
FRC: Functional residual capacity
FRI: Functional respiratory imaging
FVC: Forced vital capacity
ICC: Intra-class correlation coefficient
iRaw: Airway resistance per lung lobe
iVaw: Airway volume per lung lobe
MEF_25 − 75_: Mean expiratory flow between 25 and 75% of FVC
MEF_25_: Maximal expiratory flow where 25% of FVC remains to be expired
PEF: Peak expiratory flow
TLC: Total lung capacity
