# Supplementary material for: Exploring the link between a novel approach for computer aided lung sound analysis and imaging biomarkers: a cross-sectional study
Source: Respir Res. 2024 Apr 24;25:177. doi: 10.1186/s12931-024-02810-5 (PMC11044477; doi:10.1186/s12931-024-02810-5)
Supplement: Supplementary file 1 — Supplementary Material 1 [file 12931_2024_2810_MOESM1_ESM.docx]

**Exploring the link between a novel approach for Computer Aided Lung Sound Analysis and imaging biomarkers: a cross-sectional study.**

**Authors**

Eline Lauwers, Toon Stas, Ian McLane, Annemiek Snoeckx, Kim Van Hoorenbeeck, Wilfried De Backer, Kris Ides, Jan Steckel, Stijn Verhulst

**Online Data Supplement**

1. **Supplemental methods**
   1. CT settings

Unenhanced chest CTs were acquired by a GE VCT LightSpeed scanner using the following scanning protocol:

- rotation time: 0.6 sec
- detector coverage: 40 mm
- helical thickness: 0.625 mm
- pitch and speed: 1.375:1 and 55 mm/s
- tube voltage: 100 kV
- tube current: variable between 10 and 200 mAs
- noise index: 45
  1. Functional Respiratory Imaging

The reconstruction of the lung lobes and bronchial tree are performed in the commercially available software package, Mimics (Materialise NV, Belgium). Images are reconstructed at 0.3 mm interval using a lung filter. The resulting data set has 700-1200 images with a pixel size of 0.4-0.65 mm^2^. Lung volumes are segmented using a HU threshold of [-1024; -400], followed by a region growing operation to select only relevant voxels. The fissure lines are manually identified to separate the lung lobes. The bronchial tree, i.e. intraluminal air, is automatically segmented up to a point where no distinction can be made between intraluminal and alveolar air, which is down to a level of airways with a diameter of 1-2 mm. A typical airway model includes 5-10 generations, depending mainly on the disease state of the individual patient. More generations will be identified in patients with cystic fibrosis with extensive bronchiectasis. Air trapping can be determined through segmentation based on Hounsfield unit (HU) thresholds of [-1024; -850] performed on the FRC scan.

Functionality is added to the static segmented images by applying computational fluid dynamics (CFD) methods to characterize airway resistance. The airway models are converted into a computational grid in order to solve the Navier-Stokes flow equations numerically, using commercial software packages (Ansys Inc., Canonsburg, PA, USA).  During the CFD calculations, the outflow to each lobe is adjusted iteratively for each patient to match the internal flow distributions obtained from the segmentation of the CT scans. More specifically, the internal airflow distribution is derived from the relative lung expansion from FRC to TLC. These proportions are the boundary conditions for the steady state CFD analysis, considering a constant flow. The flow is driven by the pressure difference between the trachea, as pressure inlet, and the outlets of the airway branches. Only distal airway outlets can be considered since alveolar zones cannot be segmented. Subsequently, airway resistance is defined as the total pressure drop over an airway divided by the flow rate through that airway.

- 1. Automated CALSA

The recordings were acquired using a digital stethoscope (Thinklabs One, Thinklabs Medical LLC) with a 32-bit resolution and a sampling rate of 44.1 kHz. As shown in Figure E1, the analysis consisted of several processing blocks: (1) preprocessing, (2) motion artifact detection, (3) denoising, (4) wavelet packet decomposition to separate discontinuous adventitious sounds, (5) respiratory cycle extraction and (6) crackle peak detection. This process has been described extensively by McLane et al [1]. In summary, the signal was first low-pass filtered at 4 kHz and resampled to 8 kHz to simplify processing and remove unnecessary information. Also, motion artifacts presented as short-time, broad-band energy bursts were removed from the signal. A deep learning regression network trained and validated on simulated lung sounds was applied to decrease the unwanted noise levels. Discontinuous adventitious lung sounds (DAS) were separated from pulmonary vesicular sounds (PVS) using a wavelet packet transform-based stationary non-stationary filter. Next, respiratory cycle phases were identified from the PVS signal using the Hilbert transform for envelope extraction and recording-specific thresholds. The signal power was calculated from the PVS signal during in- and expiration to obtain the expiration-to-inspiration (E/I) power ratio for different frequency bands: 100-200 Hz, 200-400 Hz, 400-800 Hz and 800-1600 Hz. The approach to identify crackles was similar to the extraction of the respiratory phases in the PVS signal, i.e. the Hilbert transform was used to calculate the signal envelope of the DAS signal and the crackle peaks were detected using a recording-specific threshold. All CALSA analyses were performed in MATLAB (The MathWorks, Inc.).

**
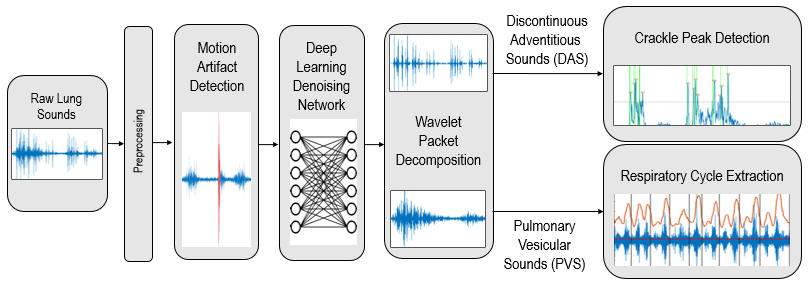
Figure E1.** Signal processing workflow. This figure was adapted from McLane et al [1].

1. **Supplemental results:**
   1. Interobserver variability analysis

**Table E1.** Interobserver variability analysis of the CF-CT scoring method

|  | **Absolute agreement** | | **Consistency** | |
| --- | --- | --- | --- | --- |
|  | ICC | P value | ICC | P value |
| Total score | 0.78 [-0.09; 0.93] | < 0.001 | 0.90 [0.81; 0.95] | < 0.001 |
| Bronchiectasis | 0.91 [0.82; 0.96] | < 0.001 | 0.93 [0.86; 0.96] | < 0.001 |
| Mucous plugging | 0.65 [-0.19; 0.87] | < 0.001 | 0.83 [0.68; 0.91] | < 0.001 |
| Bronchial wall thickness | 0.41 [-0.20; 0.72] | 0.001 | 0.63 [0.30; 0.80] | 0.001 |
| Parenchyma | 0.76 [-0.10; 0.92] | < 0.001 | 0.89 [0.79; 0.94] | < 0.001 |
| Air trapping | 0.84 [0.69; 0.91] | < 0.001 | 0.83 [0.69; 0.91] | < 0.001 |

**Figures E2-7.** Bland-Altman plots to analyse the agreement between the two observers, EL and AS. The scores represent the sum of all lobes, with the lingual considered as a separate lobe: bronchiectasis (0-72), bronchial wall thickening (0-54), mucus plugging (0-36), parenchymal abnormalities (0-54), air trapping (0-27), and total score (0-243).


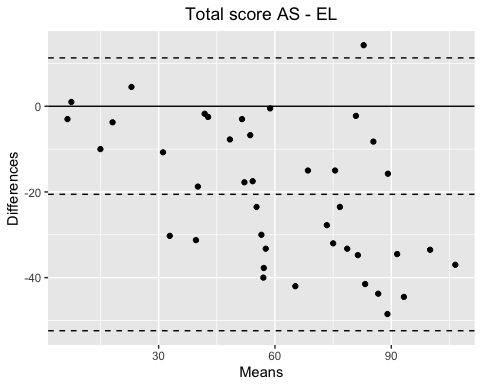

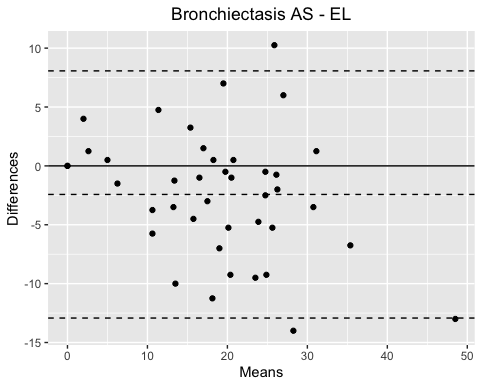


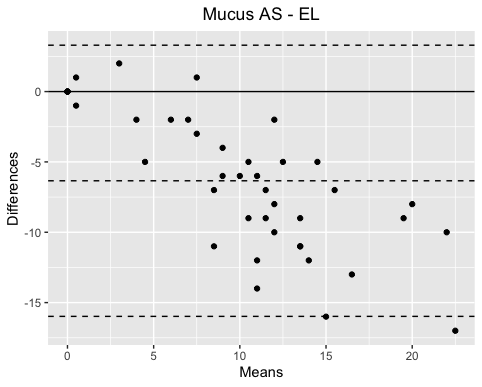

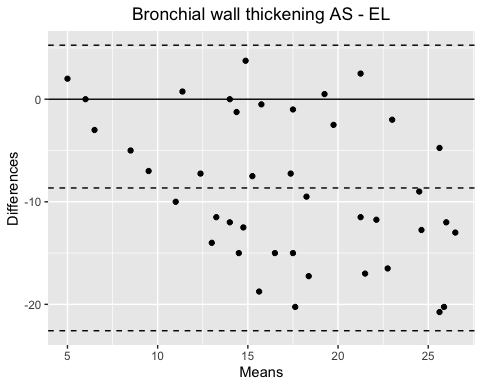


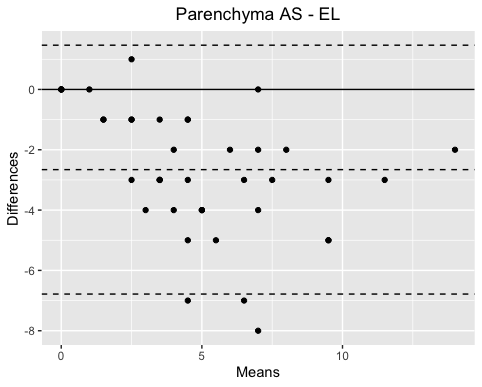

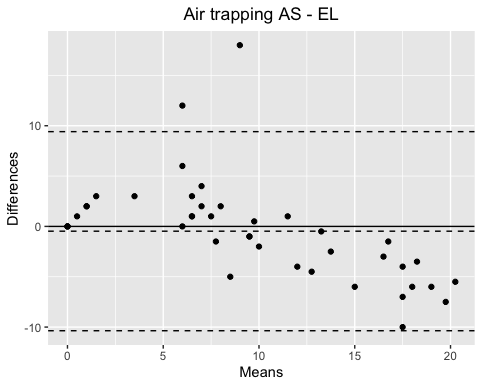


**References**

1. Mclane IM, Lauwers E, Stas T, Busch-Vishniac I, Ides K, Verhulst S, et al. Comprehensive Analysis System for Automated Respiratory Cycle Segmentation and Crackle Peak Detection. IEEE Journal of Biomedical and Health Informatics. 2021;:1–1.
